# Sulfamic acid incorporated HKUST-1: a highly active catalyst and efficient adsorbent†

**DOI:** 10.1039/d0ra01063d

**Published:** 2020-04-20

**Authors:** Mahmoud M. Kaid, Ahmed Gebreil, Soheir A. El-Hakam, Awad. I. Ahmed, Amr Awad Ibrahim

**Affiliations:** Chemistry Department, Faculty of Science, Mansoura University Mansoura Egypt amr_awad@mans.edu.eg; Department of Chemistry, Virginia Commonwealth University Richmond VA 23284-2006 USA aamohammed@vcu.edu; Nile Higher Institutes of Engineering and Technology El-Mansoura Egypt

## Abstract

Herein we introduce an effective approach for incorporating sulfamic acid (SA) into HKUST-1. The synthesized materials have been characterized using XRD, XPS, BET, FT-IR, SEM, EDX and TEM. The X-ray diffraction pattern of SA@HKUST-1 is analogous to that of parent HKUST-1 in line shape and *d*-spacing, proving that chemical modification could be obtained without damage to structural solidity. The XPS spectra confirmed successful sulfonation, due to the single S 2p peak being attributable to SO_3_H groups at 168 eV. Catalytic efficiency was studied for 7-hydroxy-4-methyl coumarin and 3,4-dihydropyrimidinone synthesis and it was found to be highly dependent on the amount of SA loaded over HKUST-1. Moreover, the adsorptive removal activity of some common organic and inorganic pollutants from water has been studied. To fully understand the adsorption process, the effects of initial dye concentration, pH of solution, adsorbent dosage, contact time and temperature on the adsorption process were successfully studied. Under the optimum conditions 10 wt% SA@HKUST-1 was able to reach the maximum adsorption capacity for Pb^2+^ (298 mg g^−1^) and Malachite green (290 mg g^−1^). Hopefully, this will facilitate research on improving the prospective use of MOFs for future applications.

## Abstract 2

Herein we introduce an effective approach for incorporating sulfamic acid (SA) into HKUST-1.

## Graphical abstract

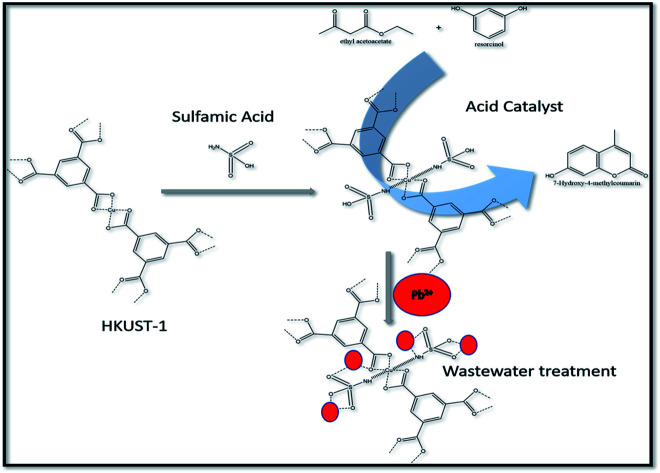

## Introduction

The last two decades have seen enormous expansion in the synthesis, characterization, and study of the chemical and physical properties of materials known as metal–organic frameworks *i.e.* MOFs. These materials are constructed by reticular synthesis in which inorganic and organic units are connected by strong bonds, creating an open crystalline frame with regular porosity.^1–3^

Post-synthetic modification “PSM” of MOFs has become an efficient method for modifying the activity of the pores.^4^ Simple loading or impregnation, reflux, and autoclave treatment are widely applied methods for PSM of MOFs. The high chemical and thermal stability of many MOFs has made them adjustable to metal-complex functionalization and PSM, which extend their applications to water-containing processes and promote their efficiency in catalytic conversion.^5–7^ The catalytic activity of varied MOFs has been demonstrated for reactions such as catalytic transformations, hydrogenation, polymerization, cyanosilylation,^8–10^ as well as for isomerization and enantioselective separations.^11,12^

Although sulfamic acid “SA” has strong Brønsted acidity, it has a relatively poor surface area which restricts its application as solid-acid catalysts.^13–15^ Therefore, materials with an ultra-high surface area are used as supports to improve the distribution of SA and forming an effective heterogeneous catalyst.

Coumarins are chemically familiar as 1,2-benzopyranone and quite available in plants were identified firstly as an oxygen heterocycle in 1820's.^16^ Their distinguished biological, therapeutic and pharmacological efficiencies *e.g.* antimicrobial and anti-inflammatory,^17,18^ antioxidant and antibacterial activities,^19^ made them materials of interest in medicinal and organic chemists.^20,21^ 7-Hydroxy-4-methyl coumarin was widely applied for SW and pulsed operation as an efficient laser dye, fluorescent brightener, fluorometric detection of enzymatic activity, initiating material for an insecticide preparation, a precursor for substituted coumarin derivatives such as furanocoumarins and a lot of others analytical reagents.^21,22^ Various methods were applied to syntheses of coumarin and its derivatives *e.g.* Pechmann reaction,^23^ Reformatsky, Wittig^24^ and Knoevenagel reaction.^25^ Among these processes, Pechmann is the widely applied reaction for coumarin synthesis which depends on using the concentrated H_2_SO_4_ as the catalyst^23^ which not only hazardous and non-regenerable material but also neutralized producing by-products, hence increases environmental pollution. Therefore, the conventional acid catalysts (H_2_SO_4_, *p*-toluene sulfonic acid, POCl_3_, trifluoroacetic acid) must be replaced by an eco-friendly and green solid catalyst. Heterogeneous catalysts have been applied to eliminate this problem, for example, zeolite H-BEA,^23^ dipyridine copper chloride and Amberlyst-15,^26^ montmorillonite clay,^25^ Nafoin/silica composites Nafion-H catalyst,^27^ W/ZrO_2_ acid catalyst,^28^ Wells–Dawson heteropolyacid^29^ and other solid acids.^30,31^

Recently, there is great interest in synthesis and studying the biological activities of 3,4-dihydropyrimidinones (DHPMs) and their derivatives including antitumor, antibacterial, antiarrhythmic activity, antiviral, anti-inflammatory, antihypertensive and antifungal activities.^32–35^ Moreover, DHPMs exhibited a pharmacological and therapeutic effect similar to that of 1,4-dihydropyridines (DHPs) modulators for calcium cavity of the nefidine.^35,36^

Nowadays, the modern chemical synthesis interested in the green processes which include using efficient, reusable and cost-effective catalysts, biodegradable, solvent-free conditions and non-toxic fluid like water. Solvent-free reactions have a lot of advantages including energy consumption, cost savings, atom economy and decreased reaction times which are good for the environment as well as being acceptable to the industry.^37^

Water purification considered an important field of research since toxic metals in the water environment can be accumulated in living organisms. The contaminant level of Pb(ii) and Cd(ii) in drinking water shouldn't exceed 10 and 3 μg L^−1^, respectively as recommended by the World Health Organization (WHO). Dye materials used in plastics industries, paper, cosmetics, leather, printing, pharmaceutical, painting, textile and food^38–40^ are one of the most abundant chemical pollutants of the water. Basic dyes such as Malachite green (MG), Rhodamine B (RhB) and Methylene blue (MB) *etc.* with high intensity of colors decrease the sunlight penetration in the aquatic life and hence may affect the photosynthetic efficiency and maybe also toxic due to the presence of aromatics, metals, *etc.*^41–43^ Adsorption has more superiority than other techniques in the purification of fuels and water, removal of hazardous materials and storage of gases such as methane and hydrogen because it can occur at low temperatures so requires lesser energy and hence of comparatively lower cost.^44,45^

Every year MOFs gaining interest in removing hazards component from water.^46–48^ Despite, HKUST-1 gaining interest in the gas phase adsorption, there is little information about its application in removing hazards components from aqueous solutions.

In the present work, we will take advantage of the post-synthetic modification of the HKUST-1 with strong Brønsted acid species like sulfamic acid to develop highly efficient and stable material, which is able to catalysis the formation of 7-hydroxy-4-methyl coumarin and 3,4-dihydropyrimidinones that were found to be dependent on the acid strength, by introducing sulfamic acid which increases the ratio of the Brønsted acid sites to the Lewis acid sites. Moreover, the introduction of new groups like NH_2_ and S=O to the HKUST-1, increase the electrostatic interactions to the cations and organic pollutant in the contaminated water. On another hand, the incorporation of the sulfamic acid enhances the stability of the HKUST-1 which is proved by the excellent reusability and regeneration ability. At present and to the best of our knowledge, there is no use of SA@HKUST-1 as catalyst and adsorbent in the literature.

## Materials and methods

CuCl_2_·2H_2_O, 1,3,5-tricarboxylic acid benzene (H_3_BTC), were obtained from Alfa-Aeser Co. Malachite green (MG) and lead nitrate were supplied from Sigma-Aldrich chemicals Co. Sulfamic acid (SA), *N*,*N*-dimethylformamide “DMF”, ethanol, ethyl acetoacetate, benzaldehyde, urea resorcinol, and lead nitrate are all with analytical grades and were used without any further purification.

### Preparation of HKUST-1

The synthesis of HKUST-1 was slightly modified from the original method.^49,50^ Solid mixtures of 7.24 mmol, 1.234 g CuCl_2_·2H_2_O and 4.48 mmol, 0.9414 g H_3_BTC were dissolved in a mixture of DMF, ethanol, and water (12 : 16 : 8 mL, respectively). After vigorous stirring for about 30 minutes at room temperature, then the homogeneous solution has been transferred into a 100 mL Teflon-lined autoclave and placed in an oven at 85 °C for 24 h. After cooling gradually to 25 °C, the product was filtered and centrifuged three times with DMF and then the solvent was exchanged by ethanol (total amount 30 mL). The product was then dried under vacuum at 80 °C for 6 hours, yielding 1.427 g of HKUST-1 and stored in a desiccator to prevent water adsorption onto the catalyst surface.

### Preparation of SA@HKUST-1 composite

The as-prepared HKUST-1 was activated under vacuum at 120 °C for 2 h in order to remove the coordinated water. Subsequently, 1 g of activated HKUST-1 was dispersed in 20 mL absolute ethanol using ultrasonic for 1 h. The different wt% sulfamic acid (3, 5, 10, 15, 20, 30, 40 and 60 wt%) also activated for 2 h and dissolved in 10 mL absolute ethanol, then added dropwise to the dispersed HKUST-1 with continuous stirring at room temperature. After vigorous stirring, the resultant mixture was transferred into a 100 mL round flask and refluxed at 100 °C for 12 h. The samples were cooled down slowly to room temperature, left to settle down overnight, then removed from the mother liquor, washed two times with ethanol, dried under vacuum at 80 °C for 6 h and finally, thermally treated in an air muffle furnace at 150 °C to yield the wt% SA@HKUST-1 composites in the form of bluish-green color.

### Catalytic activity

#### Synthesis of 7-hydroxy-4-methyl coumarin

In a typical reaction, 2.5 mL of ethyl acetoacetate (20 mmol) was mixed with 1.1 g resorcinol (10 mmol) in a 25 mL round flask immersed in an oil bath. After that, 0.05 g HKUST-1 or SA@HKUST-1 composite previously activated in a vacuum oven for 2 h at 120 °C was added to the flask and the reaction mixture was refluxed for 2 h at 120 °C. After the reaction has been completed, the catalyst was separated by simple filtration and the resultant hot filtrate was precipitated when poured over crushed ice and stirred vigorously for about 10 minutes. The obtained crude product 7-hydroxy-4-methyl coumarin was recrystallized from ethanol, dried and characterized by FT-IR spectral analysis and the % yield of the product was calculated as follows:
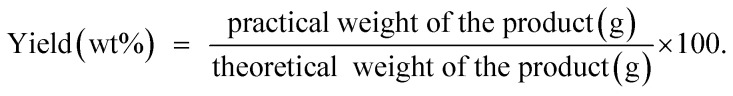


The corresponding desired product was obtained under the mild and solvent-free conditions in short reaction time, high purity, excellent yield, recyclable and green process.

#### Synthesis of 3,4-dihydropyrimidinone

Urea, ethyl acetoacetate and benzaldehyde (1.5 : 1 : 1 mmol) were refluxed at 80 °C for 15–300 minutes in the presence of HKUST-1 or SA@HKUST-1 composites as catalyst (0.05 g) pre-activated at 120 °C for 2 h, the reaction was observed by thin-layer chromatography (TLC). To our pleasant results, multicomponent reactions were consumed completely to afford the product within 90 minutes. After the reaction has been completed, the reaction mixture was filtered to separate the catalyst and the filtrate collected over crushed ice to form 3,4-dihydropyrimidinones. The product well washed with hot dist. H_2_O to remove the excess urea and dried at 80 °C. Finally, the desired solid product was recrystallized from ethanol to output in a purer form. Characterization of the desired product was done using FT-IR with specific bands at 1111, 1469, 1647, 1707, 1731, 2978, 3103 and 3245 cm^−1^ which was found to be in conformity with those mentioned in the literature^35,51^ and the % yield of the product was calculated as in case of 7-hydroxy-4-methyl coumarin.

### Adsorption activity

Stock solutions of MG dye and Pb(ii) (1000 mg L^−1^ for each, assuming that *d*_solution_ = 1 mg L^−1^) were prepared by dissolving 1 g solid MG (C_23_H_25_ClN_2_, 96% pure) and Pb^2+^ (Pb(NO_3_)_2_, 99% pure) in 1 L distilled water, separately. The working solutions (25–400 mg L^−1^) of MG dye and Pb(ii) were prepared by diluting the stock solutions with dist. H_2_O. Stock solutions of MG and Pb(ii) with a concentration between 1 and 10 mg L^−1^ were used to construct the calibration curves of absorbance. For MG the solution has been scanned using the UV-visible spectrophotometer from 190–900 nm to investigate the corresponding maximum wavelength (*λ*_max_) which was found to be 617 nm for MG dye, while in the case of Pb(ii) investigation, the AAS was used. Adsorption studies were performed with a series of glass bottles (200 mL) containing 30 mg of the adsorbent (HKUST-1 or SA@HKUST-1 composites) and a 50 mL adsorbate solution with different initial concentration ranging between 25 and 400 mg L^−1^. The pH of MG and Pb(ii) solutions was carefully adjusted to determine its influence on removal efficiency, using 0.1 N HCl and 0.1 N NaOH solutions by an Orion 420A pH-meter. The adsorption process was maintained for specific time intervals at 25 °C using a temperature-controlled, water-bath shaker. After the predetermined time, the suspension was centrifuged for 10 min at 4000 rpm to separate the solution from the adsorbent. The supernatant solution has been transferred to colorimetric tubes and the remaining equilibrium concentrations of MG dye were determined by comparing the UV-visible absorbance resulted at 617 nm for MG and AAS of Pb(ii) with the adequate standard calibration curves. The following equation has been applied at equilibrium to calculate the specific adsorbed amount of MG and Pb(ii);
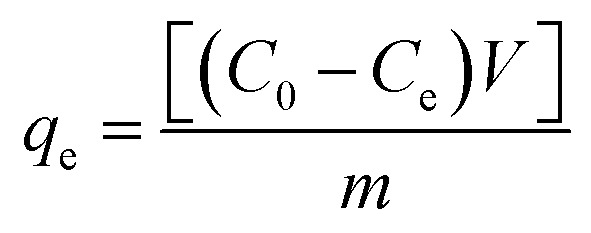
where *q*_e_ in mg g^−1^ is the adsorption capacity of the adsorbent per unit mass at equilibrium; *C*_0_ and *C*_e_ in mg L^−1^ are the initial and equilibrium concentration of adsorbate, respectively, *m* (g) is the adsorbent dosage and *V* is the volume in L of the aqueous media per.^52^ The kinetic studies have been carried out using 30 mg of the adsorbent, 50 mL of adsorbate solution with different initial concentration, pH 7 for MG and pH 5 for Pb(ii) at different ambient temperatures of 25, 35, 45 and 60 °C (to investigate the temperature effect in the removal of MG from aqueous media). Hint: to study an individual parameter, one variable has been varied, while the other parameters kept constant as discussed later herein.

### Characterization

The prepared samples were characterized using sets of different techniques. The mesoporous structure *i.e.* the bulk crystallinity of parent and modified HKUST-1 were obtained using powder X-ray diffraction (Bruker, with Cu-Kα radiation) with a D2 Phase diffractometer and were conducted at high angle 2*θ*° from 4 to 70 under the following conditions; step size of 0.02, K-alpha = 1.54 Å, 30 mA, 40 kV and scan step time of 0.80 second. SEM images of the HKUST-1 and SA@HKUST-1 composite were obtained using a scanning electron microscope (Jeol JSM-6510). To characterize the sample better, analysis of the nitrogen and sulfur contents of the SA@HKUST-1 catalyst was done using EDX analysis. The XPS data were internally calibrated, fixing the binding energy of C 1s at 284.6 eV. FT-IR analysis used effectively in proving the existence of functional groups has been recorded on a MATTSON FT-IR-5000S spectrophotometer using a KBr pellets. To well analyzed the structures, we employed transition electron microscopy (TEM, Jeol JEM-2100). For TEM micrographs, the synthesized sample was suspended using an ultrasonic apparatus in absolute ethanol for 20 min until the powder well dispersed through the solvent, then few drops of the dispersed particles were loaded on a fresh grid of carbon-coated copper. Nitrogen physisorption isotherms were collected at 77 K and pore size distributions were derived from the desorption isotherms to investigate the porous properties and pore structure of HKUS-1 and SA@HKUST-1 composites. Before the BET measurement, samples were activated at 120 °C under vacuum for 2 h. The Brunauer–Emmett–Teller (BET) equation was applied to calculate the specific surface area.

The total acidity of the synthesized catalysts has been measured according to the well-known potentiometric titration technique.^53,54^ In brief, 0.05 g solid catalyst has been firstly activated under vacuum at 100 °C for 3 h, then suspended using an ultrasonic apparatus in 15 mL acetonitrile for 15 minutes. After vigorously stirring for 6 h, The suspension was titrated at rate of 0.006 mL min^−1^ against 0.025 N *n*-butylamine diluted by acetonitrile. The electrode potential alteration was recorded using an Orion Digital Model and the strength of the acid sites and their total number were determined and reported later.

FT-IR spectral analysis of the adsorbed pyridine was used to detect the existing sites of Brønsted and Lewis acids on the catalyst surfaces. In brief, about 0.05 g of the prepared catalysts were degassed at 150 °C for 3 h under vacuum and left cooling again to 25 °C. 200 mL dry pyridine has been placed in a 250 mL beaker and then transferred into the vacuum furnace and the catalysts were maintained there for 20 days under these conditions. After this period, the samples were activated to vaporize the excess physisorbed pyridine and then investigated using a MATTSON FT-IR-5000S spectrometer at 1400–1700 cm^−1^ after mixing up the sample with KBr (0.0005 : 0.01 g, respectively).

## Results and discussion

**Fig. 1:**
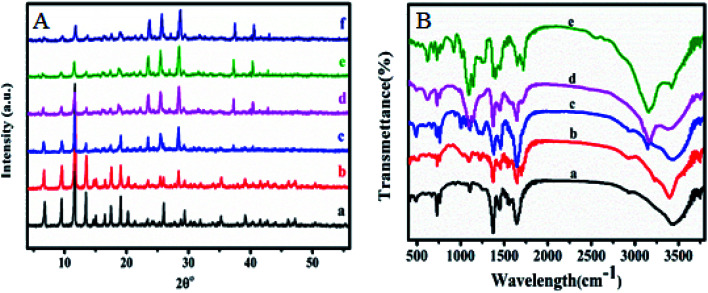
(A) X-ray diffraction patterns and (B) FT-IR spectral analysis of (a) HKUST-1, (b) 5% SA@HKUST-1, (c) 10% SA@HKUST-1, (d) 20% SA@HKUST-1, (e) 40% SA@HKUST-1 and (f) 60% SA@HKUST-1.

X-ray diffraction was performed to confirm the crystallinity of the HKUST-1. Fig. 1A shows the XRD pattern for HKUST-1 and different loading of SA on the surface of HKUST-1. The diffraction peaks around at 2*θ* of 6.83, 9.53, 11.67, 13.45, 17.49, 19.07, 20.23 and 29.37° characteristic to HKUST-1 are presented for all the prepared samples and were all consistent with the as reported by the literature.^49,50^ The Powder XRD measurements of modified SA@HKUST-1 were very analogous inline shape and *d*-spacing but with lower intensities compared to that of pure HKUST-1 indicating that there is no destruction for the as-prepared MOF after the PSM process, which may be due to the incorporation of SA by ethanol and the removal of water molecules from the HKUST-1 which clearly enhanced the stability of the modified HKUST-1.^55^ The main peaks appeared at 2*θ* of 23.47, 25.55, 28.57, 37.37 and 40.43° indicated the well incorporation of SA into the structure of the prepared MOF, also the intensity of these peaks increased with increasing of SA as reported in Fig. 1A.

The FT-IR spectroscopy of HKUST-1 and SA@HKUST-1 composites were reported in Fig. 1B. The strong absorption peak at 1640 cm^−1^ in conjunction with the disappearance of strong peaks at 1760–1690 cm^−1^ approved the deprotonation of carboxyl groups (–COOH) in 1,3,5-benzenetricarboxylate upon the interaction with copper ions confirming the formation of MOF which also proved by the softy peaks around 480 and 730 cm^−1^ spectral range that mainly due to the in-plane and out plane bending modes of (COO^−^) carboxylate group.^49,50^ The sharp peaks around 767 cm^−1^ and between 1380–1600 cm^−1^ related to C–H bending vibrations and C=C stretching vibration of the benzene ring, respectively;^56^ indicating the presence of H_3_BTC linker (organic ligand) in the synthesized MOF. The moderate peak at 727 cm^−1^ featured for the (Cu–O) vibration mode, indicated that HKUST-1 was indeed synthesized. The broad peaks in the spectral domain of 3000–3600 likely because of the acidic OH of the carboxyl (–COOH) group or crystalline water. The amino group of SA exhibits two average absorptions, one at 3155 cm^−1^ while the other around 3430 cm^−1^. These peaks represent the symmetric and asymmetric N–H stretching modes, respectively. This importantly demonstrates that the amino group is available and chemically reactive. Moreover, in all modified HKUST-1 with SA, new peaks appeared at 1280, 1150 cm^−1^ assigned to the asymmetric and symmetric stretching modes of O=S=O, respectively. Also, the weak band due to (S–O) stretching vibration appeared around 619 cm^−1^,^57^ increases with increasing the SA content.

**Fig. 2:**
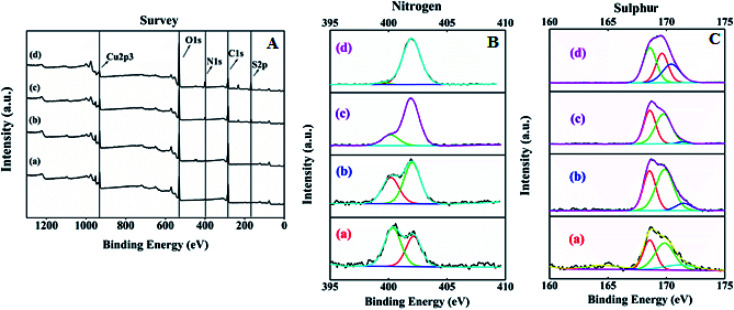
(A) X-ray photoelectron spectroscopy (XPS) of (a) HKUST-1, (b) 10% SA@HKUST-1, (c) 20% SA@HKUST-1, (d) 40% SA@HKUST-1. (B) and (C) are nitrogen and sulphur XPS of (a) 10% SA@HKUST-1, (b) 20% SA@HKUST-1, (c) 40% SA@HKUST-1 and (d) 60% SA@HKUST-1.

The surface elemental composition and states of the resultant SA@HKUST-1 were tested using XPS. The XPS spectrum of SA@HKUST-1 showed five peaks centered at 166.7 eV (S 2p), 284.9 eV (C 1s), 400.5 eV (N 1s) and 531.8 eV (O 1s) and 932.3 eV (Cu 2p) as shown in Fig. 2A. High-resolution N 1s spectrum in Fig. 2B shows two peaks attributed to N=C (deprotonated (≈91%) amine group) and –C–O–HN/–NH–SO_3_H (protonated (≈9%) amine group) at 400.2 and 402.1 eV, respectively. The surface XPS analysis of the N 1s region for 60 wt% SA@HKUST-1 shows one symmetric peak at 402 ± 0.1 eV suggesting a single nitrogen environment on the surface, which is assigned to the –C–O–HN/–NH–SO_3_H group (protonated (≈9%) amine group). One can note the absence of deprotonated amino group should be observed around 400 eV which can be easily explained by the high loadings of SA. Furthermore, the S 2p spectrum represented in Fig. 2C showed three peaks attributed to the S=O and S–O/N–S groups at 171.5, 169.8 and 168.6 eV, respectively. On the 10 wt% SA@HKUST-1, a minor S 2p signal at 168.6 eV and N 1s signal at 400–400.2 is allocated to a small fraction of thiol and amino groups in comparison with those of high loadings. Table 1S† demonstrates the surface elemental ratio and showed an obvious increase in both S 2p and N 1s % as wt% of sulfamic acid increases and these results are in agreement with the corresponding XRD and FT-IR spectrum. High-resolution O 1s, C 1s and Cu 2p XPS signals are presented in Fig. 1S.† The O spectrum showed two peaks attributed to the C=O and C–OH/C–O–C groups at 531.8 and 533.4 eV, respectively. Furthermore, there were three peaks for the high-resolution C 1s spectrum assigned to the C–C/C=C, C–N, and C=O/N–C=O groups, noticed at 284.8, 286.4 and 288.6 eV, respectively. The peaks observed at 932.3 and 952.5 eV were characteristic of Cu 2p_3/2_ and Cu 2p_1/2_, respectively.

**Fig. 3:**
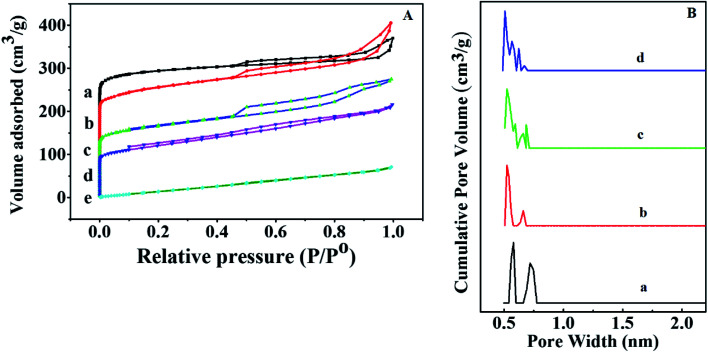
(A) N_2_ adsorption and desorption isotherms and (B) pore volume distribution of (a) HKUST-1, (b) 10% SA@HKUST-1, (c) 20% SA@HKUST-1, (d) 40% SA@HKUST-1 and (e) 60% SA@HKUST-1.

Nitrogen adsorption–desorption isotherms were measured with liquid nitrogen at 77 K and shown in Fig. 3A and B. HKUST-1 with exceptionally high surface area and ordered nano-pores are exploited to homogeneously disperse and encapsulate a considerable amount of sulfamic acid (SA). The Brunauer–Emmett–Teller (BET) equation was applied to calculate the specific surface area was found to be 1250 m^2^ g^−1^ for HKUST-1, which is in agreement with earlier reports.^58–60^ Modification with SA exhibited a significant decrease in the corresponding BET surface areas in addition to the pore volume with the increase in the SA content as recorded in Table 1. This could be due to the pore blockage resulted from the encapsulation of SA nanoparticles inside the pores. According to the International Centre for Theoretical and Applied Chemistry classification, these shapes indicate that they are Type II isotherm. One can notice that 60% wtSA@HKUST-1 exhibited a plateau with N_2_ adsorption almost zero and there were no hysteresis loops observed under relatively high pressure, indicating its non-porous structure.

**Table 1:** The surface area and acidity measurements of HKUST-1 and wt% SA@HKUST-1 catalysts obtained from BET measurements, pyridine adsorption and potentiometric titration curves

Sample	*S*_BET_	Pore volume	Initial potential (*E*_i_) in mV	No. of acid sites in m_eqv._ g^−1^ (×10^20^)	(B/L) ratio
Pure HKUST-1	1250	0.45	183.9	1.98	0.955
5% SA@HKUST-1	—	—	266.5	2.71	1.397
10% SA@HKUST-1	1068	0.39	310.5	3.55	2.275
20% SA@HKUST-1	692.7	0.25	389.1	3.98	2.819
40% SA@HKUST-1	513.6	0.178	412.9	4.32	3.639
60% SA@HKUST-1	36.7	0.015	403.4	4.09	3.528

**Fig. 4:**
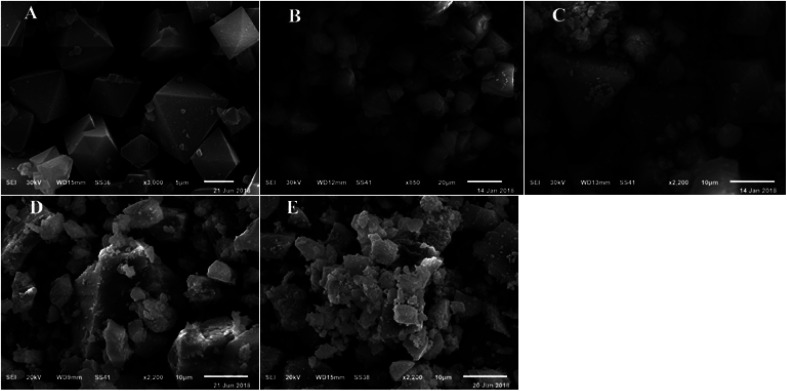
SEM micrographs of (A) HKUST-1, (B) 10% SA@HKUST-1, (C) 20% SA@HKUST-1, (D) 40% SA@HKUST-1 and (E) 60% SA@HKUST-1.

SEM micrograph for HKUST-1 confirmed that a crystalline framework was completely achieved and matched the fully octahedral shape with a relatively rough surface (Fig. 4). Also, the SEM images for different wt% SA@HKUST-1 composites are analogous to that of HKUST-1, proving that the structural solidity of pure HKUST-1 was maintained after chemical modification by SA which is uniformly distributed across the MOF crystals. The EDX analysis spectra (Fig. 2S†) confirmed the successful loading of N and S after modification of HKUST-1 with SA. TEM images of SA@HKUST-1 with different SA weight percent loading, indicated homogeneously distributed of SA on the surface of HKUST-1 (Fig. 2S†).

The acidity of HKUST-1 and SA@HKUST-1 were examined by non-aqueous potentiometric titration. The initial electrode potential (*E*_i_) obtained from this technique, displays the maximum acid strength of the catalyst surface while the overall number of acid sites per gram solid catalyst (m_eq_ g^−1^) has been obtained from the domain where a plateau is extended.^61,62^ The acidity measurements resulted from the potentiometric titration curves (Fig. 3S(A) and (B)†) of HKUST-1 and different wt% SA@HKUST-1 catalysts were recorded in Table 1. The results demonstrated the high initial and total acidity of SA@HKUST-1 compared to that of pure HKUST-1. The addition of SA resulted in a significant increase in the overall number of acid sites and acid strength till attain the maximum at 40 wt% SA@HKUST-1 (*E*_i_ = 412.9 mV), but beyond that slightly decrease was observed, which may be due to the agglomeration of SA on the HKUST-1 surface.^32^

Pyridine adsorption was used to investigate the sites of Brønsted and Lewis acids. Fig. 3S(C)† shows the FT-IR spectral analysis of the adsorbed pyridine over pure and modified HKUST-1 with different percentages of SA. All the investigated samples showed strong spectral peaks at 1543 cm^−1^ and 1437 cm^−1^ which corresponded to the adsorbed pyridine by the Brønsted acid sites and the coordinated pyridine *i.e.* Lewis acid sites, respectively. The data of Brønsted (B) to Lewis (L) sites ratio was recorded at Table 1, pure HKUST-1 holds mainly Lewis acid sites identified at 1438 cm^−1^ and resulted from cupper with coordinately unsaturated sites (CUS) than mild Brønsted sites resulted from carboxylic acid of the organic linkers (H_3_BTC) that are not attached to cupper. While SA@HKUST-1 composite holds both sites of Lewis and Brønsted acid with varied ratios based on the percentage content of SA. The Brønsted over Lewis (B/L) sites ratio, as expected significantly increased with increasing up SA percent till it reaches the maximum at 40 wt% SA@HKUST-1 while beyond that it's slightly reduced again as shown in Fig. 3S(D)† which may be due to agglomeration of SA on the surface as confirmed by SEM and TEM analysis.

### Catalytic activity

#### Synthesis of 7-hydroxy-4-methylcoumarin

**Fig. 5:**
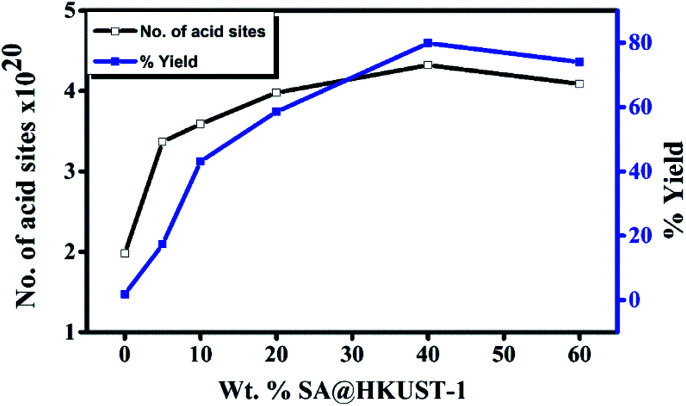
Weight % of SA@HKUST-1 against overall number of acid sites and % yield of 7-hydroxy-4-methyl coumarin.

The progress of the reaction was examined with ethyl acetoacetate and resorcinol under conditions of catalyst-free and solvent-free, even after 5 hours, no product was obtained under these conditions (Table 2S,† entry 1). The catalytic cyclization of ethyl acetoacetate and resorcinol was studied over HKUST-1 and SA@HKUST-1 catalysts to elucidate the effect of total number of acid sites on the conversion of resorcinol as shown in Fig. 5. For the as-prepared HKUST-1 only 2% was achieved after 2 h, increasing the content of SA increases the activity to 43% by only 10% SA, while more addition of SA resulted in increase of the activity to 80% for the 40 wt% SA which demonstrates the effective role of Brønsted sites due to SA and also proved that the coumarin syntheses is mainly controlled by Brønsted acid sites. These Brønsted acid sites activate ethyl acetoacetate through protonation of the ester carbonyl, hence easiness nucleophilic attack of resorcinol with ester. SA as a catalyst also showed high yield conversion (Table 2S,† entry 9), even though, the advantages of SA@HKUST-1 compared to SA and other catalysts are: (i) the ease of catalyst separation from the reaction mixture, (ii) high yield conversion (iii) high thermal stability of the catalyst, (iv) straight forward synthetic route (v) reuse of catalyst is possible and (vi) environmentally safe and eco-friendly.

To optimize the conditions, synthesis of 7-hydroxy-4-methyl coumarin from catalytic condensation of ethyl acetoacetate and resorcinol was performed by varying the molar ratios of the reactants, reaction time, catalyst weight and the reaction temperature. The results at Table 3S† showed that the adequate yield (80%) for syntheses of 7-hydroxy-4-methyl coumarin could be achieved using two equivalent of ethyl acetoacetate with one equivalent of resorcinol and 0.07 g of 40 wt% SA@HKUST-1 after 2 h at 120 °C. The product structure was confirmed by FT-IR analysis (Fig. 4S†).

After the reaction of ethyl acetoacetate with resorcinol has been completed, the SA@HKUST-1 catalyst was separated by simple filtration, washed with ethanol several times (total amount 50 mL), dried and reactivated at 120 °C in a vacuum furnace for 3 h and recycled by adding to a fresh reaction mixture. Even after five runs of catalyst applied and recovered, only 8% decrease in percentage yield was observed as shown in Fig. 7. The representative FT-IR spectral analysis of the reused SA@HKUST-1 confirmed that the catalyst maintains its structure compared to a fresh one as shown in Fig. 5S.† There are no remarkable changes in the FT-IR peaks of the SA@HKUST-1 catalyst, indicating that the HKUST-1 can well stabilize SA for several durabilities. Table 2S† shows the comparison between our catalyst and other catalysts from the literature, the results confirm that our catalyst considers one the best catalyst used for this reaction.^23,27,28,30,63^

### Biginelli reaction (synthesis of 3,4-dihydropyrimidinone)

**Fig. 6:**
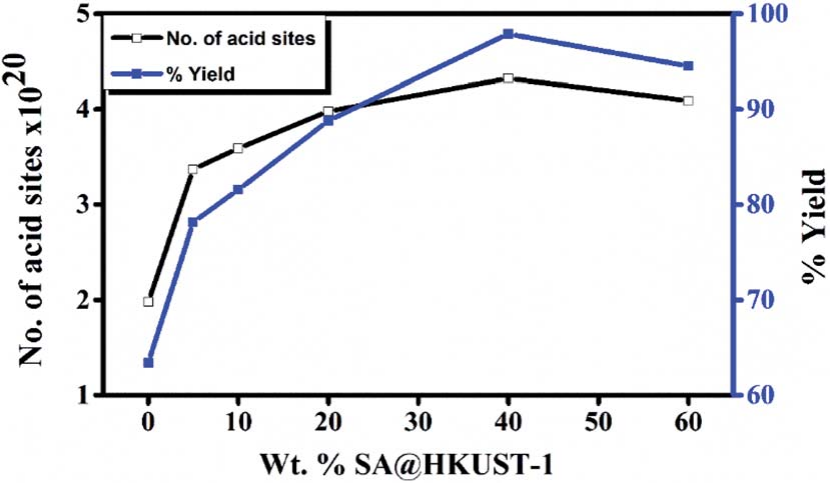
Weight % of SA@HKUST-1 against overall number of acid sites and % yield 3,4-dihydropyrimidinone.

The reaction was performed in different solvents (ethanol, isopropanol, DMF, H_2_O and under solvent-free conditions) to optimize the conditions, even after 5 h, the reaction shows no activity in absence of the catalyst at room temperature, when the temperature reached to 100 °C, only 17% yield was obtained after 5 h under conditions of catalyst-free and solvent-free (Table 4S,† entries 1 and 2). HKUST-1 shows good activity for this reaction (63% after 2 h) and this may be due to the presence of Lewis acid site, the addition of SA which increases the ratio of Brønsted to Lewis acid sites, significantly increases the yield reaching 98% for 40 wt% SA@HKUST-1. Fig. 6 shows the effect of a total number of acid sites and the catalytic activity, the activity increases with increasing of the total number of the acid sites which confirms the reaction depends on both the total number of acid sites and B/L ratio (Table 1). For the control experiment and to detect the catalyst efficiency, we have monitored the same reaction using the individual component of the catalyst; CuCl_2_·2H_2_O as Lewis acid, H_3_BTC and SA as Brønsted acids and the same amount of HKUST-1 without SA were applied for the synthesis of 3,4-dihydropyrimidinone under the same conditions. As recorded at Table 4S,† only 63% produced for more than 2 h using activated HKUST-1, 74% using SA, 52% using H_3_BTC acid and 30% using CuCl_2_, confirming the co-operative effect between HKUST-1 and SA, also the % yield increased with increasing B/L acid sites ratio.

For more investigation of the role of the combination between SA and HKUST-1, 40 wt% SA@HKUST-1 composite has been subjected to further detailed study of 3,4-dihydropyrimidinones synthesis. The percentage yield of the desired product under the different molar ratio of the reactant components were studied by varying the limiting reactant *i.e.* urea, while the other two reactants *i.e.* ethyl acetoacetate and benzaldehyde are held constant (Table 5S,† entries 1–3). The results showed that (1.5 : 1 : 1 mmol; urea, ethyl acetoacetate and benzaldehyde, respectively) produced the highest percentage yield in lower time. The optimum condition of the other operating parameter *e.g.* temperature, time and catalyst dose were summarized in Table 5S.†

The product structure was confirmed by FT-IR analysis. Its IR spectrum displayed strong absorption peaks at 1685–1591 cm^−1^ (C=O, ring), 1718–1702 cm^−1^ (C=O, acetyl) and 3292–3227 cm^−1^ (N–H) as shown in Fig. 6S.† Moreover, benzene deformed bands were noticed also in the predictable wave number areas which were found to be in conformity with those mentioned in the literature.^35,51^

**Fig. 7:**
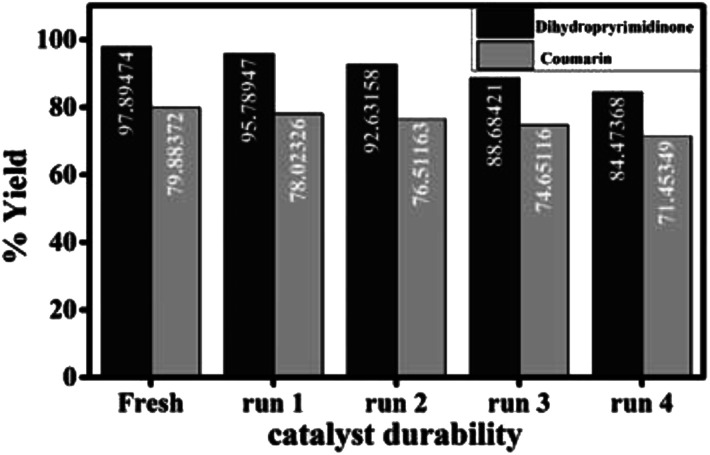
Durability of the catalyst for the synthesis of 3,4-dihydropyrimidinone and 7-hydroxy-4-methyl coumarin.

The durability (reusability) of the catalyst is critical in practical applications. The solid catalyst easily separated from the reaction mixture, washed several times with ethanol, dried, activated at 120 °C and recycled by adding to a fresh reaction mixture for four times with a slight loss in catalytic activity which may be due to inevitable loss of the catalyst weight during the process of collection and washing (Fig. 7).

The reaction mechanism may begin through the formation of acylimine intermediate by the interaction between urea and the aldehyde. Subsequently, β-ketoester enolate was added to the acylimine, followed by dehydration and cyclization *via* SA@HKUST-1 catalyst, giving the desired 3,4-dihydropyrimidinone.^35,64^

Comparison studies with previously reported catalysts applied for the synthesis of 3,4-dihydropyrimidinone was recorded in Table 4S,† entries 12–22. The study showed that the catalyst efficiency of SA@HKUST-1 was so higher in comparison with several previously reported catalysts *e.g.* DCC,^35^ cellulose sulfuric acid,^65^ BSA,^35^ AlCl_3_,^35^ silica sulfuric acid,^65^*p*-toluene sulfonic acid,^65^ Nafion NR-50,^66^ KSF^67^ and ionic liquid.^68^ The comparison study not only depended on the percentage yield of the desired product but also the condition required to optimize the reaction.

### Adsorptive removal of MG dye and Pb(ii) using HKUST-1 and SA@HKUST-1 composites

MG dye and Pb(ii) both usually present in positive forms; therefore, they can easily undergo favorable electrostatic interactions with the MOF adsorbents having a negatively charged framework (surface). HKUST-1 and SA@HKUST-1 composites were successfully used to adsorb MG and Pb(ii) which are both cationic but structurally and size difference. The adsorption efficiency of HKUST-1 greatly improved when combined with some co-ordination groups *e.g.* the thiol and amino functional groups, which has a strong affinity for capturing the potentially toxic metals ions. The small incorporated SA particles instead of water molecules of [Cu_3_(BTC)_2_(H_2_O)_3_]_*n*_ has a higher activity than those loaded on the surface. This helps using of small acid loadings for efficient adsorption removal of pollutants.

### Screening of adsorbents

**Fig. 8:**
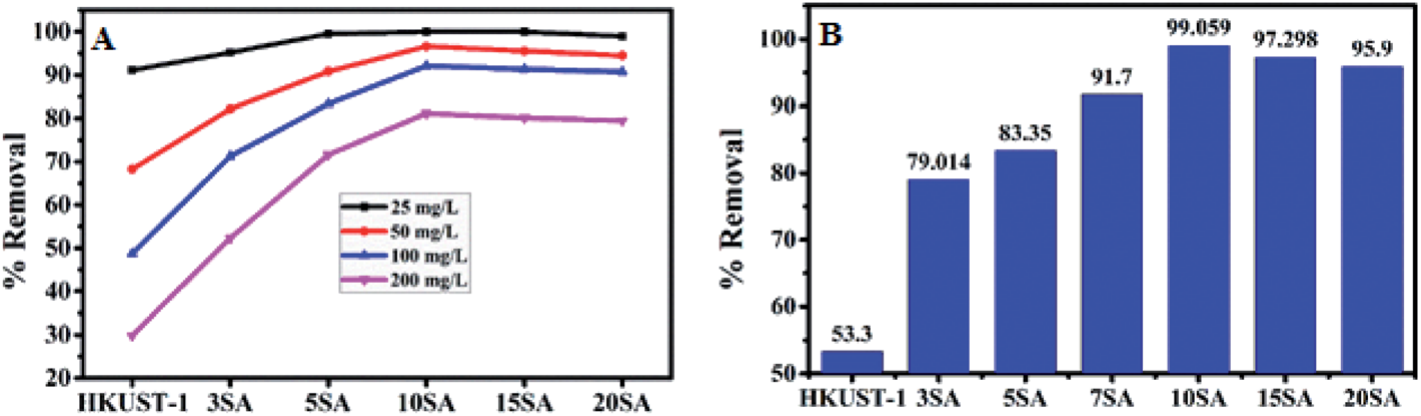
The adsorption efficiency (% removal) for (A) MG and (B) 100 mg L^−1^ of Pb(ii) on the parent HKUST-1 and different % SA@HKUST-1 composites.

Initially, HKUST-1, 3% SA@HKUST-1, 5% SA@HKUST-1, 10% SA@HKUST-1, 15% SA@HKUST-1 and 20% SA@HKUST-1 composites have been screened for MG dye and Pb(ii) adsorption. The screening was investigated using 30 mg of adsorbent, 50 mL initial adsorbate concentration at 25 °C for 24 h in a temp. controlled, water bath shaker at a shaking rate of 120 rpm. At equilibrium, the results obtained for the adsorption efficiencies (% removal) for the parent HKUST-1 and different SA@HKUST-1 composites are shown in Fig. 8. Under the same conditions, the results showed that there are no obvious differences in the efficiency adsorption between different adsorbents for lower dye concentrations, but the efficiency sharply increases at higher concentrations in order of increasing loading of SA till reach maximum for 10 wt% SA@HKUST-1 composite and then decrease again for higher loadings of SA which may be due to aggregations on the surface and hence, decreasing the pores available for adsorption. Among six types of adsorbents, 10 wt% SA@HKUST-1 showed the maximum efficiency for the uptake of both MG and Pb(ii). Therefore, 10 wt% SA@HKUST-1 composite has been subjected to further detailed study of the adsorption process. In conclusion, much better adsorption efficiency was observed after functionalizing HKUST-1 by grafting with SA, even though the pore size and porosity of pure HKUST-1 decreased after grafting with SA. That is maybe due to the N on MG dyes that can form a hydrogen bond with the H on the amino, sulfate and carboxyl groups of SA@HKUST-1 composites, in addition to the electrostatic attraction between the cationic adsorbates and negatively charged of the adsorbent surface.

### Detection of single operating parameter

The individual influences of solution pH, adsorption time, initial dye concentration, adsorbent dose and temperature on the uptake efficiency of MG and Pb(ii) by SA@HKUST-1 has been studied to detect the optimum range of each single operating factors. In order to achieve this, we noticed the influence resulted from changes in a single parameter while the other operating parameters were maintained constant. The studied range and the optimum value of each parameter for the adsorption of MG and Pb(ii) using 10 wt% of SA@HKUST-1 were shown in Fig. 7S and 8S† and the results summarized in Table 2. Under optimum conditions, 10 wt% SA@HKUST-1 showed maximum adsorption efficiency (92%, 99%) for 100 mg L^−1^ of MG and Pb(ii), respectively.

**Table 2:** Detection of the optimum value of each parameter for MG dye and Pb(ii) adsorption using 10 wt% SA@HKUST-1 composites

Operating parameter	Test range	Optimum value	
		MG	Pb
pH	2–12	7	5
Contact time (h)	0–24	6	0.75
Adsorbent dosage (g L^−1^)	0.2–2	0.6	0.6
Initial dye concentration (mg L^−1^)	25–400	100	100
Temperature (°C)	25–60	25	Nil

### Adsorption isotherms

For a solid–liquid system, one of the important physicochemical studies in the characterization of sorption behavior is the equilibrium isotherm of sorption which used to characterize the experimental adsorption data. The Langmuir isotherm is the most applied adsorption model and aspects monolayer sorption behavior on the adsorbent surface which contains a homogeneous and definite number of active sites.^69,70^ The model generally expressed in the well-known form represented in the following equation as:
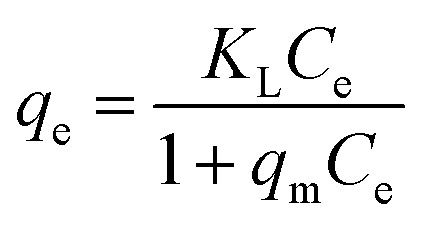


A linear form of this model expression is:
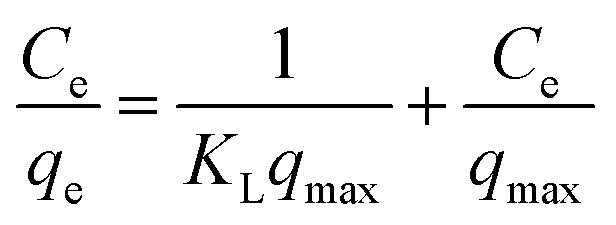
where *C*_e_ (mg L^−1^) is the equilibrium concentration of the adsorbed molecule; *q*_e_ (mg g^−1^) is the adsorption capacity of the adsorbent at equilibrium per unit mass of adsorbent; *q*_max_ (mg g^−1^) indicates the theoretical sorption capacity and *K*_L_ in L mg^−1^ is the Langmuir sorption isotherm constant. One of the most important descriptions of the Langmuir isotherm equations is the dimensionless separation factor (*R*_L_), represented in the equation as:^41,42,71^
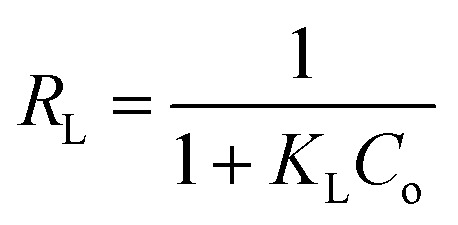
where *K*_L_ in L mg^−1^ is the Langmuir sorption constant and *C*_0_ in mg L^−1^ is the initial concentration of the adsorbed molecule. The *R*_L_ values describe the isotherm type to be either irreversible (*R*_L_ = 0), linear (*R*_L_ = 1), unfavorable (*R*_L_ > 1) or favorable (0 < *R*_L_ < 1). On the other hand, the well-known heterogeneous systems; Freundlich isotherm does not take into account the formation of monolayer but multilayer and non-ideal adsorption. The Freundlich isotherm model represented by the well-known logarithmic form which expressed in the following equation:^42,71^*q*_e_ = *K*_F_*C*_e_^1/*n*^

A linear form of this expression can be represented as follow:ln *q*_e_ = (1/*n*)ln *C*_e_ + ln *K*_F_where *n* (g L^−1^) and *K*_F_ (mg^1−1/*n*^ L^1/*n*^ g^−1^) are the Freundlich sorption isotherm exponent and constant. *K*_F_ describes the adsorption or distribution coefficient giving an indication of the definite quantity of the adsorbed molecule on an adsorbent surface at equilibrium while *n* indicates the favorability of the sorption isotherm and measure the surface heterogeneity or sorption intensity. The process is more heterogeneous when the value of 1/*n* gets nearer to zero. A value of 1/*n* lower than unity is indicative of Langmuir isotherm while 1/*n* above unity indicating of co-operative sorption.^41,69^ Langmuir and Freundlich sorption isotherms for MG dye and Pb(ii) using SA@HKUST-1 composite as adsorbent are seen in Fig. 9S.† The correlation factor coefficients, *R*^2^ and isotherm parameters are calculated and recorded in Table 3. The Langmuir model with *R*^2^ factor of 0.9985 and 0.9988 for MG and Pb, respectively, represents the accurate fit of the experimental data and more acceptable than that of Freundlich isotherm with *R*^2^ of 0.8690 and 0.8969 for MG and Pb(ii), respectively. It gives an indication of the monolayer sorption of both MG and Pb(ii) on the homogeneous surface of SA@HKUST-1 adsorbent. The maximum monolayer capacity obtained from the Langmuir model for the uptake of MG and Pb is found to be 290.7 and 298.51 mg g^−1^, respectively. Moreover, the dimensionless separation constant *R*_L_ values between 0.009–0.137 for MG and 0.0047–0.053 for Pb(ii) indicates the favorability of the sorption process for both dyes, also the value of 1/*n* between 0 and 1 also indicates the high sorption intensity as shown in Table 3.

The thermodynamic parameters for the adsorption of MG dye and Pb(ii) using 10 wt% SA@HKUST-1

**Table:** 

Langmuir isotherm constants				
	*K*_L_ (L g^−1^)	*q*_m_ (mg g^−1^)	*R*_L_	*R* ^2^
MG	0.251	290.69	(0.009–0.137)	0.9985
Pb(ii)	0.708	298.51	(0.0047–0.053)	0.9988

**Table:** 

Freundlich isotherm constants			
	*K*_F_ (mg g^−1^)	*n*	*R* ^2^
MG	83.73	3.78	0.869
Pb(ii)	137.58	5.43	0.8969

### Regeneration of 10 wt% SA@HKUST-1

One of the important key factors in water treatment is the reusability (durability) of the adsorbents which are good for the environment from the green chemistry point of view as well as being acceptable to the industry *i.e.* cost-saving and atom economy. Hence, we further examined the durability of 10 wt% SA/HKUST-1. In this study, hot ethanol was adopted as the eluent solvent for the regeneration of the used adsorbent. As shown in Fig. 10S,† 10 wt% SA@HKUST-1 almost maintained their initial percent removal (the efficiency decreased only 10% for MG dye) over four repeated runs. From the FT-IR spectral analysis of the fourth reused SA@HKUST-1 in comparison with that of fresh one (Fig. 11S†), there is no obvious change that happened to groups of SA@HKUST-1 during the recovering *i.e.* showed the same spectral peaks. In conclusion, 10 wt% SA@HKUST-1 adsorbent was easily recovered after the dye uptake process and reused at least four times.

A comparison study was made between the results achieved in this work through 10 wt% SA@HKUST-1 with those obtained by other adsorbents reported previously in the literature *e.g.* Bentonite,^72^ CNF aerogel,^73^ almond gum,^74^ CuS-NRs-AC,^75^ ZnS:Cu-NP-AC,^76^ CO_2_-activated carbon (CAC).^77^ The results demonstrated that SA@HKUST-1 among other various adsorbents mentioned above and reported at Table 6S,† behaves as an effective adsorbent for adsorption of MG dye and Pb(ii) with respect to maximum adsorption capacity (mg g^−1^).

## Conclusion

HKUST-1 and SA@HKUST-1 composites were prepared successfully, characterized using sets of different techniques *e.g.* XRD, XPS, BET, FT-IR, SEM, EDX analysis and TEM. Post synthetic modification of parent HKUST-1 with SA sharply increased the surface acidity and catalytic activity. This paper offers SA@HKUST-1 with various advantages such as easily prepared in larger amounts, high catalytic activity, straightforward synthetic route under mild conditions, high thermal stability, simple handling, easy recovery without losing its activity which makes it an eco-friendly, green and efficient catalyst. The catalytic results of SA@HKUST-1 composite catalyzing 7-hydroxy-4-methylcoumarin and 3,4-dihydropyrimidinone, in comparison with that of parent HKUST-1 or pure SA showed a higher yield of the desired product in a lower time. Among various wt% of SA loadings, 4 0 wt% SA@HKUST-1 showed the highest catalytic activity and acidity. Even after the 4^th^ run, the SA@HKUST-1 catalyst still well maintained nearly the initial catalytic activity, proving that SA has been effectively incorporated in the framework of HKUST-1. HKUST-1 and SA@HKUST-1 composites also were applied in adsorption of MG dye and Pb(ii). We found that the removal efficiency was high for lower MG concentration of both the parent HKUST-1 and SA@HKUST-1 composite with fast kinetics. But, in case of higher concentration of MG, the efficiency of HKUST-1 was reduced below 50% of the original value, while SA@HKUST-1 composite still removed the MG and Pb(ii) up to >85% which indicates the better adsorptive ability of SA@HKUST-1 composite compared to the parent MOF alone. The adsorption performance has been greatly improved at pH 7 for MG and pH 5 for Pb(ii) with removal efficiency reached approximately 92% and 99% from 100 mg L^−1^ of MG and Pb(ii) solution, respectively. The adsorption equilibrium on the prepared materials were reached after 6 h for MG and 45 min. for Pb under optimum conditions. The study of temperature change demonstrates that the sorption efficiency decreased with increasing temperature from 25 to 60 °C suggesting the exothermic nature of the sorption process. The experimental data resulted from the equilibrium studies were fitted to the Langmuir sorption model and this adsorption system follows the pseudo 2^nd^ order kinetic model. Even the fourth durability, the uptake capacity reduced only within 10% for MG dye which still considered as a high removal value. The FT-IR spectral analysis confirmed the good reusability and high structural stability of the adsorbent. Subsequently, we trust that SA@HKUST-1 composite as an adsorbent can have a promising future for environmental pollutants purification and separation.

## Conflicts of interest

There are no conflicts to declare.

## Supplementary Material

RA-010-D0RA01063D-s001
