# Supplementary material for: Sulfamic acid incorporated HKUST-1: a highly active catalyst and efficient adsorbent
Source: RSC Adv. 2020 Apr 20;10(26):15586–97. doi: 10.1039/d0ra01063d (PMC9052581; doi:10.1039/d0ra01063d)
Supplement: RA-010-D0RA01063D-s001 [file RA-010-D0RA01063D-s001.pdf]

Electronic Supplementary Material (ESI) for RSC Advances.
This journal is © The Royal Society of Chemistry 2020

Sulfamic Acid Incorporated HKUST-1: A Highly Active Catalyst and Efficient Adsorbent

Mahmoud M. Kaid[a], Ahmed Gebreil[a,c], Soheir A. El-Hakam[a], Awad. I. Ahmed[a], Amr Awad Ibrahim[a,b*]

[a] Chemistry Department, Faculty of Science, Mansoura University, Mansoura, Egypt

[b] Department of Chemistry, Virginia Commonwealth University, Richmond, VA 23284-2006, United States.

[c] Nile Higher Institutes of Engineering and Technology, El-Mansoura, Egypt

[a,c*] Corresponding authors., amr_awad@mans.edu.eg, aamohammed@vcu.edu

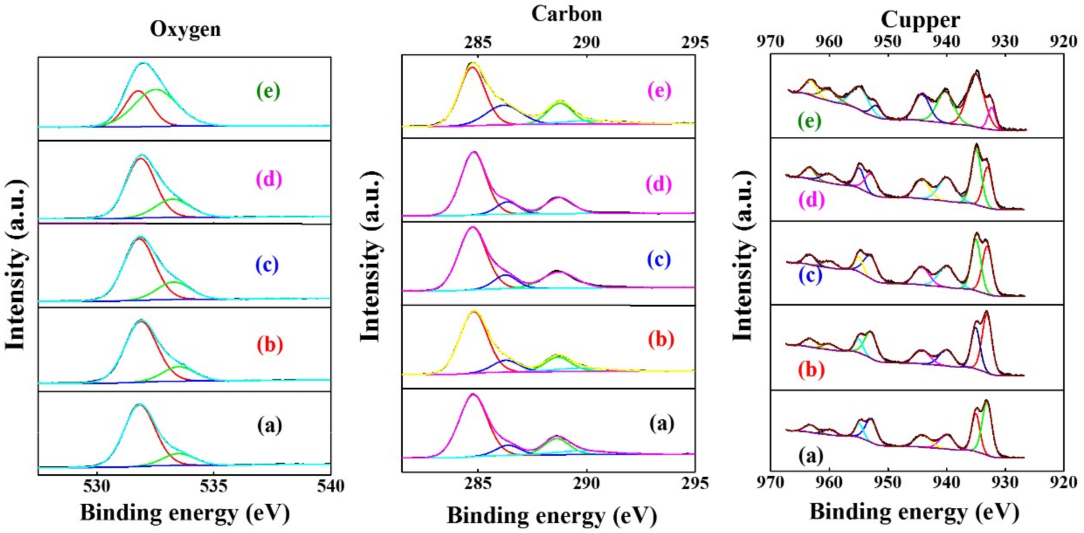

Fig. 1S: X-ray photoelectron spectroscopy (XPS) of (a) HKUST-1, (b) 10% SA@HKUST-1, (c) 20% SA@HKUST-1, (d) 40% SA@HKUST-1 and (e) 60% SA@HKUST-1.

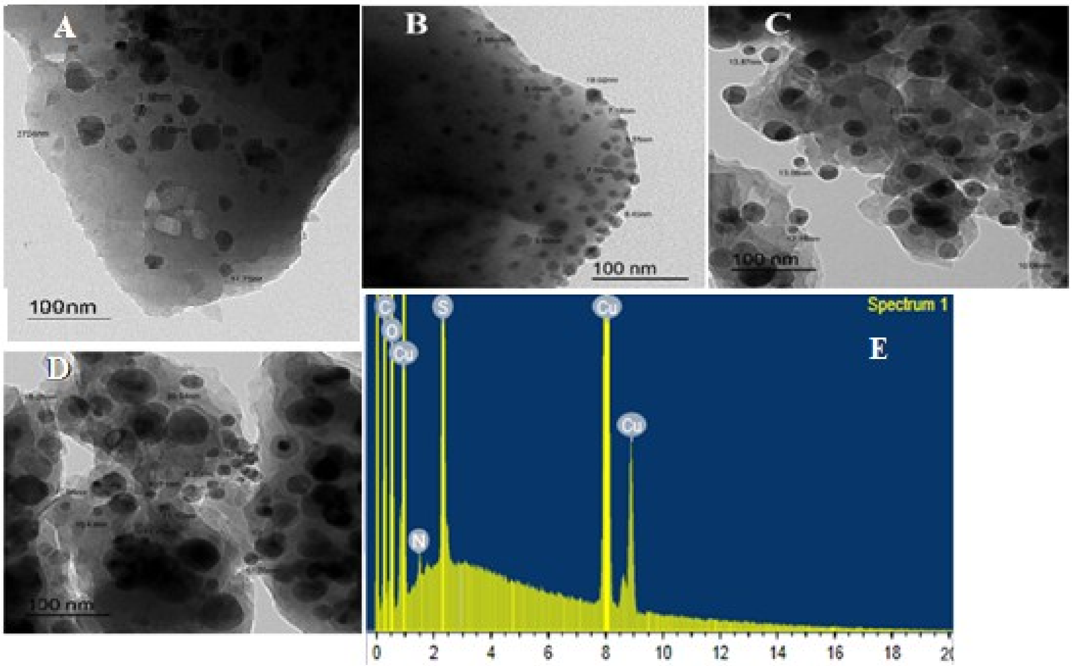

Fig. 2S: TEM images of (a) 10%SA@HKUST-1, (b) 20%SA@HKUST-1, (c) 40%SA@HKUST-1 and (d) 60%SA@HKUST-1. **(E)** EDX analysis spectra of 10%wt. SA@HKUST-1.

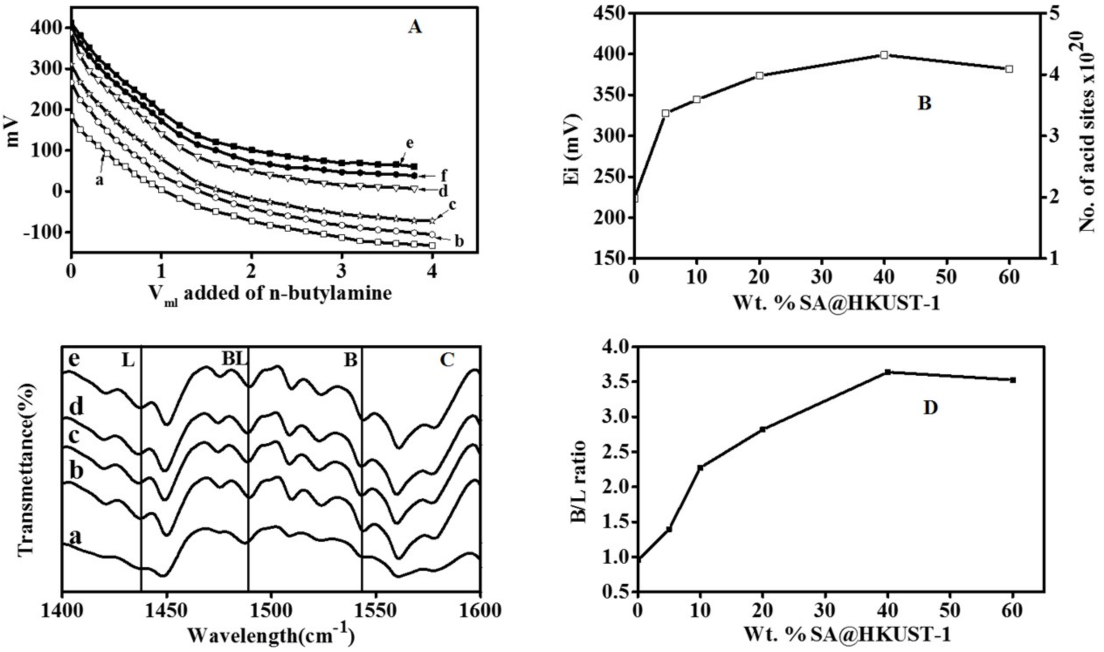

Fig. 3S: (A)Potentiometric titration curves of (a) HKUST-1, (b) 10%SA@HKUST-1, (c) 20%SA@HKUST-1, (d) 40%SA@HKUST-1 and (e) 60%SA@HKUST-1, (B) Effect of SA wt.% on the strength of acid sits, (C) FT-IR spectral analysis of chemisorbed pyridine of (a) HKUST-1, (b) 10%SA@HKUST-1, (c) 20%SA@HKUST-1, (d) 40%SA@HKUST-1 and (e) 60%SA@HKUST-1 and (D) Effect of SA wt.% on the B/L ratio

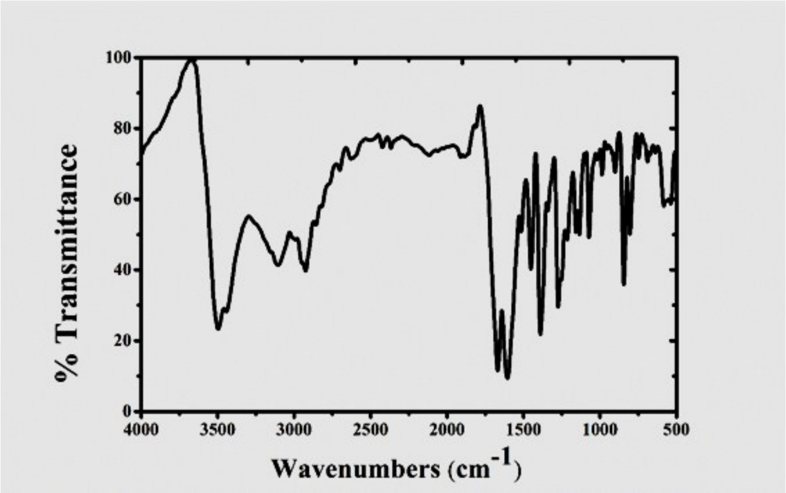

Fig. 4S: FT-IR spectral analysis of 7-hydroxy-4-methylcoumarin.

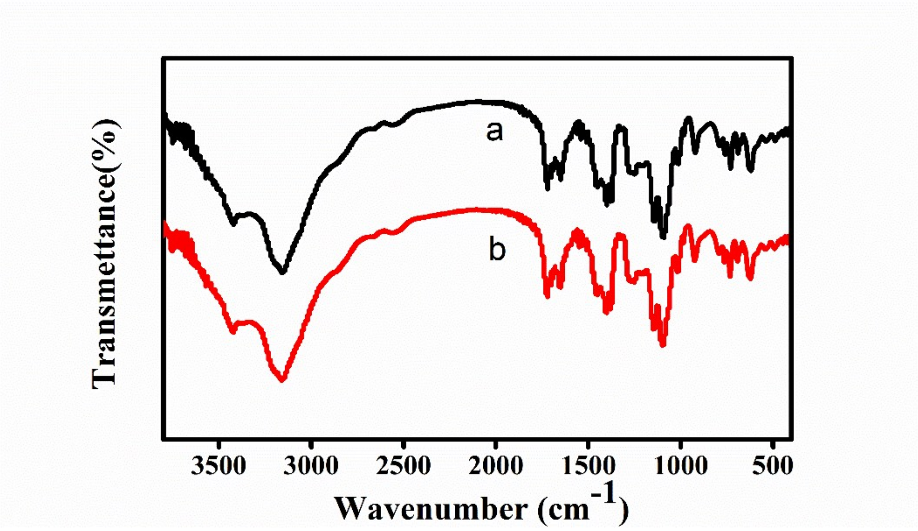

Fig. 5S: FT-IR spectral analysis of (A) fresh 40wt% SA@HKUST-1 and (B) the reused 40wt% SA@HKUST-1 after 4th run.

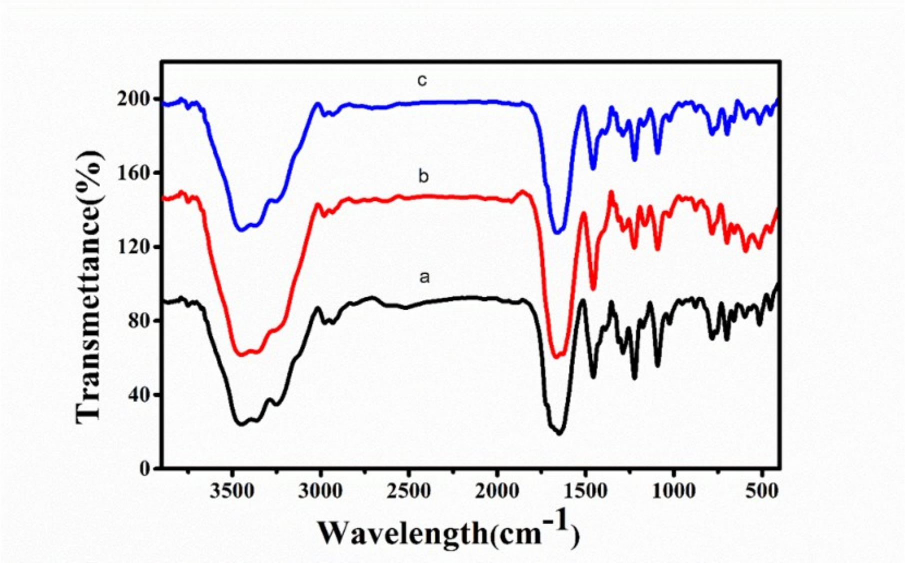

Fig. 6S: FT-IR spectral analysis of the synthesized 3,4-dihydropyrimidinone under (a) solvent free and 0.05g catalyst, (b) ethanol as solvent and 0.05g catalyst and (c) solvent free and reused catalyst.

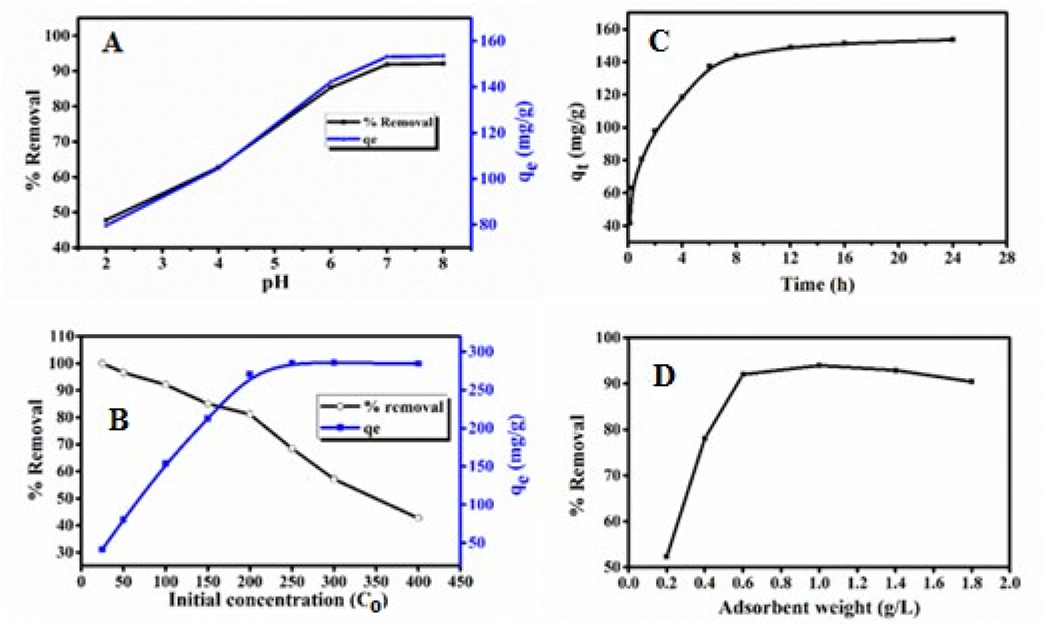

Fig. 7S: Effect of (A) pH, (B) initial dye concentration, (C) contact time and (D) adsorbent dose on the adsorption of MG dye using 10wt. % SA@HKUST-1 composite.

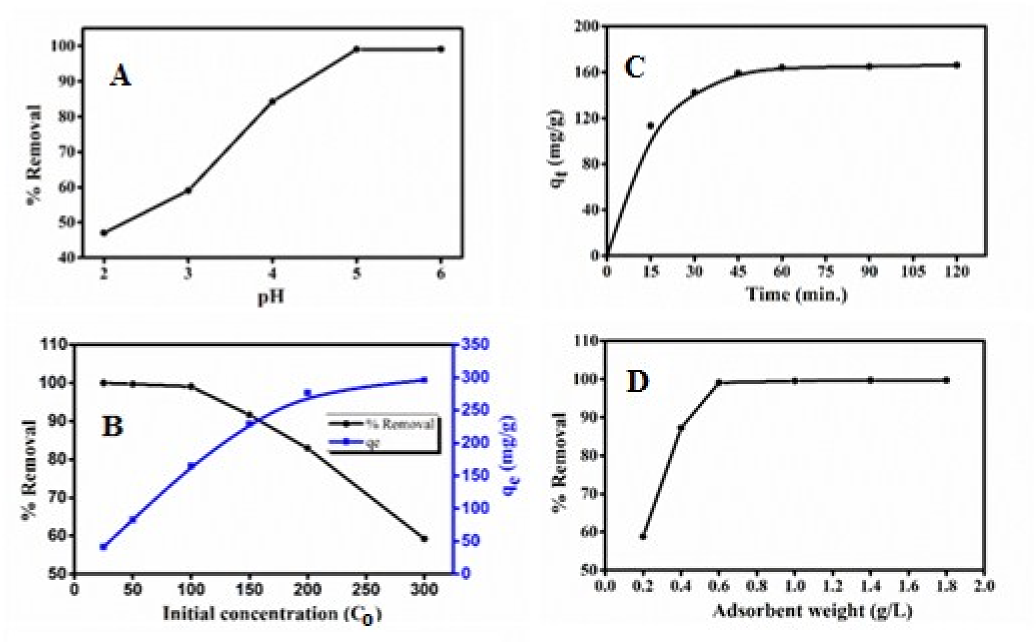

Fig. 8S: Effect of (A) pH, (B) initial dye concentration, (C) contact time and (D) adsorbent dose on the adsorption of Pb (II) using 10wt. % SA@HKUST-1 composite.

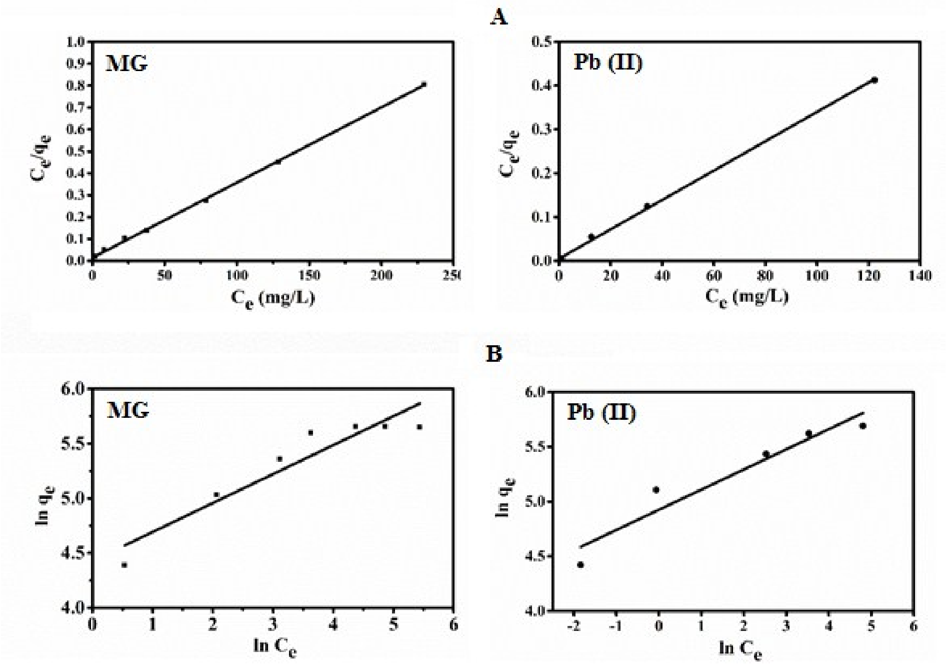

Fig. 9S: (A) Langmuir and (B) Freundlich adsorption isotherms (initial concentration 50–400 mg/L for MG and Pb, 0.03 g adsorbent, 25 °C; at optimum pH).

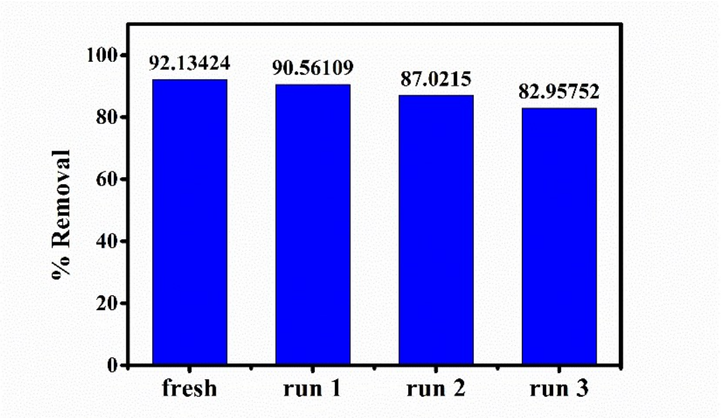

Fig. 10S: Reusability of 10wt. % SA@HKUST-1 applied in adsorption of MG dye.

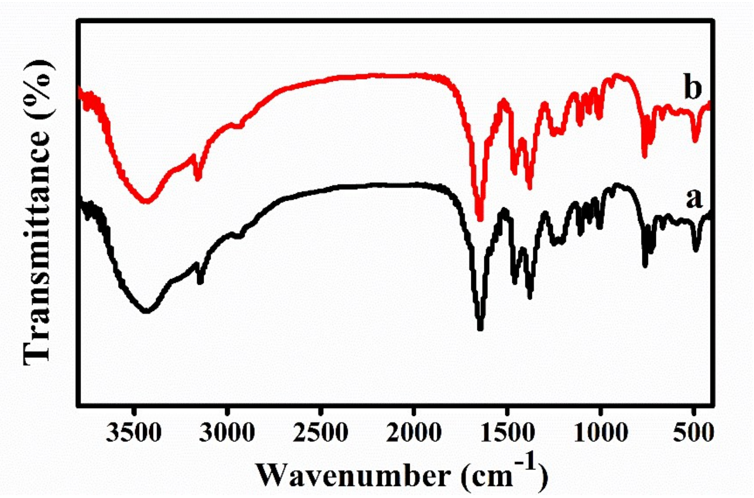

Fig. 11S: FT-IR spectra of (a) fresh 10wt. % SA@HKUST-1 and (b) reused 10wt. % SA@HKUST-1.

Table 1S: The surface elemental ratio of SA@HKUST-1.

Sample	C1s%	O1s %	Cu2p3%	N1s%	S2p%
Pure HKUST-1	66.65	28.01	5.35	0	0
5% SA@HKUST-1	63.93	28.75	4.35	2.43	1.14
10% SA@HKUST-1	64.15	28.03	4.12	1.06	1.99
20% SA@HKUST-1	59.67	30.57	4.08	2.97	2.72
40% SA@HKUST-1	52.65	33.27	3.75	5.39	4.96
60% SA@HKUST-1	33.3	43.97	2.98	9.63	10.09

Table 2S: Comparison study for synthesis of 7-hydroxy-4-methylcoumarin in presence of HKUST-1 and wt. % SA@HKUST-1 with other catalysts reported in the literature.

Entry	Catalyst	Conditions	% Yield	Ref.
1	Free	Solvent free, Reflux, 120°C, 5h	Nil	This work
2	HKUST-1	Solvent free, Reflux, 120°C, 2h	2	This work
3	10wt% SA@HKUST-1	Solvent free, Reflux, 120°C, 2h	43	This work
4	20wt% SA@HKUST-1	Solvent free, Reflux, 120°C, 2h	59	This work
5	40wt% SA@HKUST-1	Solvent free, Reflux, 120°C, 2h	80	This work
6	60wt% SA@HKUST-1	Solvent free, Reflux, 120°C, 2h	74	This work
7	$CuCl_2.2H_2O$	Solvent free, Reflux, 120°C, 2h	Nil	This work
8	H_3BTC	Solvent free, Reflux, 120°C, 2h	28	This work
9	SA	Solvent free, Reflux, 120°C, 2h	67	This work
10	ZAPO-5	Toluene,175°C, 4h,	34	[63]
11	LaZAPO-5	Toluene,175°C, 4h,	51	[63]
12	CeZAPO-5	Toluene,175°C, 4h,	57	[63]
13	H-Beta	Toluene, Reflux, 4h	71	[26]
14	Amberlyst- 15	Solvent free, 150°C, 2h	72	[26]
15	W/ZrO2	Toluene, 6h, 0.3g	80	[28]
16	Acidic ionic liquid	Solvent free, 2h	75	[30]

Table 3S: The optimum reactant molar ratio (ethyl acetoacetate: resorcinol), temperature, time and catalyst dose required for solvent free synthesis of 7-hydroxy-4-methylcoumarin using 0.05 g of 40wt. % SA@HKUST-1.

Entry	Reactant molar ratio (mmole)	Temperature (^oC)	Time (min.)	Catalyst dose (g)	% Yield
1	1:1	120°C	120	0.07	42
2	2:1	120°C	120	0.07	80
3	3:1	120°C	120	0.07	67
4	4:1	120°C	120	0.07	59
5	2:1	25°C	120	0.07	6
6	2:1	50°C	120	0.07	24
7	2:1	100°C	120	0.07	72
8	2:1	120°C	120	0.07	80
9	2:1	130°C	120	0.07	80
10	2:1	120°C	15	0.07	19
11	2:1	120°C	30	0.07	41
12	2:1	120°C	60	0.07	61
13	2:1	120°C	90	0.07	73
14	2:1	120°C	120	0.07	80
15	2:1	120°C	180	0.07	82
16	2:1	120°C	300	0.07	83
17	2:1	120°C	120	0.01	31
18	2:1	120°C	120	0.03	56
19	2:1	120°C	120	0.05	70
20	2:1	120°C	120	0.07	80
21	2:1	120°C	120	0.1	80
22	2:1	120°C	120	0.2	80

Table 4S: Comparison study of HKUST-1 and different wt% SA@HKUST-1 catalyzed synthesis of 3,4-dihydropyrimidinone with other catalysts reported in the literature.

Entry	Catalyst	Conditions	%Yield	Ref.
1	Free	Solvent free, RT, 5h	Nil	This work
2	Free	Solvent free, 100°C, 5h	17	This work
3	Non activated HKUST-1	Solvent free, Reflux, 80°C, 2h	35	This work
4	Activated HKUST-1	Solvent free, Reflux, 80°C, 2h	63	This work
5	10wt% SA@HKUST-1	Solvent free, Reflux, 80°C, 2h	81	This work
6	20wt% SA@HKUST-1	Solvent free, Reflux, 80°C, 90 min.	89	This work
7	40wt% SA@HKUST-1	Solvent free, Reflux, 80°C, 90 min.	98	This work
8	60wt% SA@HKUST-1	Solvent free, Reflux, 80°C, 90 min.	95	This work
9	$CuCl_2.2H_2O$	Solvent free, Reflux, 100°C, 2h	30	This work
10	H_3BTC	Solvent free, Reflux, 100°C, 2h	52	This work
11	SA	Solvent free, Reflux, 100°C, 2h	74	This work
12	DCC	Solvent free, 3–4h	71	[35]
13	Cellulose sulfuric acid	CH_2Cl_2	68	[65]
14	Cellulose sulfuric acid	EtOH	96	[65]
15	Cellulose sulfuric acid	MeOH	86	[65]
16	BSA	Solvent free, 2–4h	80	[35]
17	$AlCl_3$	Solvent free, 8–10h	40	[35]
18	Silica sulfuric acid	Solvent, 100°C	91	[65]
19	p-Toluene sulfonic acid	Solvent, 100°C	85	[65]
20	Nafion NR-50	3h	96	[66]
21	KSF	Solvent, 100°C	82	[67]
22	Ionic liquid	Solvent free, 100°C	86	[68]

Table 5S: The optimum reactant's molar ratio (urea: ethyl acetoacetate: benzaldehyde, respectively), temperature, time and catalyst dose required for solvent free synthesis of 3,4-dihydropyrimidinone using 0.05 g of 40wt. % SA@HKUST-1.

Entry	Reactant molar ratio (mmole)	Temperature (^{o}C)	Time (min.)	Catalyst dose (g)	% Yield
1	1:1:1	80	90	0.05	88
2	1.5:1:1	80	90	0.05	98
3	2:1:1	80	90	0.05	92
4	1.5:1:1	25	90	0.05	19
5	1.5:1:1	50	90	0.05	70
6	1.5:1:1	80	90	0.05	98
7	1.5:1:1	100	90	0.05	98
8	1.5:1:1	120	90	0.05	98
9	1.5:1:1	80	15	0.05	59
10	1.5:1:1	80	30	0.05	82
11	1.5:1:1	80	60	0.05	91
12	1.5:1:1	80	90	0.05	97
13	1.5:1:1	80	120	0.05	97
14	1.5:1:1	80	180	0.05	98
15	1.5:1:1	80	300	0.05	98
16	1.5:1:1	80	90	0.01	74
17	1.5:1:1	80	90	0.03	91
18	1.5:1:1	80	90	0.05	97
19	1.5:1:1	80	90	0.1	98
20	1.5:1:1	80	90	0.2	97
21	1.5:1:1	80	90	0.3	97

Table 6S: Comparison of the maximum monolayer adsorption q_m (mg/g) of MG dye adsorbed by 10wt% SA@HKUST-1 with other adsorbents reported in the literature.

Entry	Adsorbent	q_m (mg/g)	Ref.
6	SA/HKUST-1	291	This work
7	Bentonite	178	[72]
8	CNF aerogel	212	[73]
9	Almond gum	196	[74]
10	CuS-NRs-AC	145	[75]
11	ZnS : Cu-NP-AC	168	[76]
12	CAC	210	[77]
